# Evolution and pathogenicity of H6 avian influenza viruses isolated from Southern China during 2011 to 2017 in mice and chickens

**DOI:** 10.1038/s41598-020-76541-0

**Published:** 2020-11-25

**Authors:** Weishan Lin, Hongrui Cui, Qiaoyang Teng, Luzhao Li, Ying Shi, Xuesong Li, Jianmei Yang, Qinfang Liu, Junliang Deng, Zejun Li

**Affiliations:** 1 College of Veterinary Medicine, Sichuan Agricultural University, Chengdu, China; 2 Shanghai Veterinary Research Institute, Chinese Academy of Agricultural Sciences, Shanghai, People’s Republic of China

**Keywords:** Influenza virus, Viral evolution, Viral pathogenesis

## Abstract

H6 subtype avian influenza viruses spread widely in birds and pose potential threats to poultry and mammals, even to human beings. In this study, the evolution and pathogenicity of H6 AIVs isolated in live poultry markets from 2011 to 2017 were investigated. These H6 isolates were reassortant with other subtypes of influenza virus with increasing genomic diversity. However, no predominant genotype was found during this period. All of the H6N2 and most of the H6N6 isolates replicated efficiently in lungs of inoculated mice without prior adaptation. All of the H6N2 and two H6N6 isolates replicated efficiently in nasal turbinates of inoculated mice, which suggested the H6N2 viruses were more adaptive to the upper respiratory tract of mice than the H6N6 viruses. One of H6N2 virus caused systemic infection in one out of three inoculated mice, which indicated that H6 avian influenza virus, especially the H6N2 viruses posed a potential threat to mammals. Five H6 strains selected from different genotypes caused no clinical signs to inoculated chickens, and their replication were limited in chickens since the viruses have been detected only from a few tissues or swabs at low titers. Our study strongly suggests that the H6 avian influenza virus isolated from live poultry markets pose potential threat to mammals.

## Introduction

Avian influenza virus (AIV), belonging to influenza A virus in the family of Orthomyxoviridae, contains eight negative stranded RNA segments. Influenza A viruses are subdivided into 18 different hemagglutinin (HA) and 11 neuraminidase (NA) subtypes based on the surface proteins^1–4^. Subtype H6 is one of the widespread AIV subtypes though it is easy to be neglected for its low pathogenicity in birds^5–7^.

The H6 AIV was isolated first from a turkey in the United States in 1965^8,9^. Since then, H6 AIVs have been detected frequently from wild aquatic birds and domestic poultry throughout the world^10–12^. During 2000–2002, H6N2 AIVs were isolated from chickens from 12 different locations in California. The pathological changes observed in the early cases were primarily associated with mild respiratory infections, but yolk peritonitis had become the main feature of all the subsequent cases through 2001 and 2002^13^. In 2009, H6N1 AIV was isolated once again from turkey exhibiting typical signs associated with AIV infection in Israel^14^.

Domestic ducks act as an important reservoir for influenza viruses and have also facilitated the establishment of multiple H6 influenza virus lineages^15^. Among those, the H6N2 and H6N6 were the main epidemiological subtypes recently circulated in Southern China^6,7,15,16^. Though clinical disease caused by H6 in chicken farms has not been reported in China, H6 AIVs were isolated continually from chickens in the live poultry markets (LPMs) and might pose a huge threat on the poultry industry. Previous studies have shown that the H6 AIVs have a broad host range, and the H6 viruses might have crossed the species barrier and infected mammals, including humans, without adaptation^17,18^. In 2010, an avian-origin H6N6 swine influenza virus was isolated from sick pigs in Southern China^19^. Three years later, an H6N1 virus was isolated from a woman with flu-like symptoms in Taiwan^18^. In addition, an H6N1 virus with molecular characteristics closely related to the human isolates, was found to cause infections in dogs^20^. H6 AIVs have the potential to cross the species barrier and infect mammals, including humans^18,20^. Around 34% of H6 viruses isolated from Southern China could bind to the human-like receptor^17^. Specially, a single amino acid change from glutamine to leucine at position 226 of hemagglutinin causes a switch in receptor-binding preference from avian-like to mammalian-like^21^. In addition, H6 AIVs provided internal genes for other subtype viruses and generated novel viruses frequently, such as H5N1, H9N2 and H5N6 AIVs which were detected in humans^22–24^.

Recently, a large amount of H6 AIVs were isolated in Southern China through routine surveillance and their phylogenetic characters were analyzed comprehensively^25^. However, the pathogenicity of those H6 AIVs on chickens and mammals are unclear. In this study, the evolution and pathogenicity of 7 H6N2 and 15 H6N6 viruses, isolated from LPMs in Southern China from 2011 to 2017, were evaluated in mice and chickens.

## Materials and methods

### Virus isolation

Oropharyngeal swabs were collected at different LPMs in Hunan, Jiangsu, Zhejiang, Guangdong and Fujian provinces from 2011 to 2017. Each swab was soaked in phosphate-buffered saline (PBS) and tested by reverse transcription and polymerase chain reaction (RT-PCR) using influenza-specific HA primers for H6 subtype as described previously^26,27^. To isolate the viruses, the H6-positive samples were filtered through 0.22 μm filters and inoculated into allantoic cavity of 9-day-old specific pathogen free (SPF) embryonated chicken eggs (Beijing Merial Vital Laboratory Animal Technology Co., Ltd.,Beijing, China). The viruses were purified and propagated by 3 rounds of limiting dilution in embryonated SPF chicken eggs^28^. The viral titers were determined in SPF embryonated chicken eggs and calculated using the Reed and Muench method^29^.

### Genetic analysis and phylogenetic analysis

Viral RNAs were extracted from allantoic fluid containing H6 viruses with the QIAamp viral RNA mini kit (Qiagen, Gemany) according to the manufacturer’s instructions. The first-strand cDNAs were synthesized using reverse transcriptase M-MLV (Takara Bio Inc., Dalian, China) with universal primer for influenza A viruses (5`- AGC RAA AGC AGG-3`) following the manufacturer’s protocol. The eight gene segments of each virus were amplified by PCR with universal primers for influenza^30^. The PCR were carried out in an C1000 Thermal cycler PCR Instrument (Biorad, U.S.A.) using Premix Ex Taq version 2.0 (Takara Bio Inc., Da Lian, China) following the manufacturer's instructions. The PCR products were purified using DNA Gel Extraction Kits (Axygen, Hangzhou, China) and sequenced by GENEWIZ Biotechnology Company (Suzhou, China). The sequences were edited with Seqman module of the DNAStar package. Phylogenetic trees were generated by the distance-based neighbor-joining method using Clustal W. The reliability of the trees was assessed by bootstrap analysis with 1000 replications^31^. Reference sequences were cited from GenBank of NCBI and GISAID.

### Studies with mice

To determine the pathogenicity of theH6 AIVs in mice, each of eight 4-week-old female BALB/c mice (Beijing Merial Vital Laboratory Animal Technology Co., Ltd., Beijing, China) were anesthetized and inoculated intranasally (i.n.) with 10^6.0^ EID_50_ in 30.0 μl PBS of each virus of the 22 H6 isolates. Eight control mice were inoculated intranasally with 30.0 μl of PBS. At five days post-inoculation (dpi), three mice in each group were euthanized, and their lungs, nasal turbinates, hearts, livers, spleens and brains were collected for virus titration. Each organ was homogenized in chilled PBS and centrifuged at 2500*g* for 10 min. The viruses in the supernatants were titrated using the methods described previously^29^. The body weight and clinical signs of remaining five mice in each group were recorded until 14 dpi.

### Studies with chickens

The pathogenicity of 5 H6 viruses from different genotypes, was evaluated in SFP chickens. Each of six 4-week-old SPF chickens were inoculated i.n. with 10^6.0^EID_50_/bird of each virus in a volume of 100.0 μl, respectively. One day later, three naïve chickens were introduced into each of these isolates to evaluate the transmission of viruses among chickens. Six control birds were inoculated i.n. with the same amount of PBS. Three inoculated chickens in each group were euthanized, and their tracheas, lungs, kidneys, spleens and duodenums were collected for virus isolation at 3 dpi. Cloacal and oropharyngeal swabs were collected from the other 3 chickens at 2, 4 and 6 dpi. All of the tissues were homogenized in PBS (0.1 g per 1.0 ml), and the swabs in 1.0 ml PBS were stirred on vortex, and then all samples were centrifuged at 2500 g for 10 min. Each supernatant was collected, filtered through 0.22 μm filters, and inoculated into allantoic cavities of three 9-day-old embryonated chicken eggs. At 14dpi, blood samples were drawn from the remaining inoculated chickens and three contact chickens for detection of H6 subtype specific antibodies.

### Ethics statement and statistical analysis

All animal studies in this study were conducted in accordance to the guidelines of the Animal Care and Use Committee of Shanghai Veterinary Research Institute, and all animal studies protocols are approved by Shanghai Veterinary Research Institute (Permit number: SHVRI-Po-0120). Good living environment with sufficient food and water were available for all the animals. Comparisons of the weight changes between two groups were determined by nonparametric t-tests using GraphPad (Vision 6.0, GraphPad Prism). The differences were considered statistically significant if P values < 0.05(*), P < 0.01(**), P < 0.001(***).

## Results

### Virus isolation and identification

A total of 22 H6 AIVs were isolated from ducks and geese without any obvious clinical signs in LPMs during 2010–2017. All of viruses were propagated in SPF chicken embryonated eggs and their virus titers ranged from 10^6.3^ to 10^9.5^ EID_50_/100 μl. Full genomes of 7 H6N2 and 15 H6N6 viruses were sequenced and deposited to the NCBI database, the accession number and abbreviations of viruses used in this study have been listed in Table 1.

**Table 1:** Abbreviations used and GenBank accession numbers for H6 Avian Influenza virus isolates.

Virus	Abbreviation	Virus titer	GenBank accession No. for gene							
		(EID50/100 μl)	PB2	PB1	PA	HA	NP	NA	M	NS
A/duck/Hunan/A729-2/2011(H6N6)^a^	HN/A729	10^7.5^	MT828554	MT828845	MT829178	MT826249	MT828279	MT827972	MT827847	MT828310
A/goose/Guangdong/1268–1/2011(H6N2)	GD/1268	10^7.8^	MT828555	MT828846	MT829179	MT826250	MT828280	MT827973	MT827848	MT828311
A/goose/Guangdong/1127–1/2011(H6N2)	GD/1127	10^9.5^	MT828556	MT828847	MT829180	MT826251	MT828281	MT827974	MT827849	MT828312
A/duck/Hunan/2282–1/2011(H6N6)	HN/2282	10^8.3^	MT828557	MT828848	MT829181	MT826252	MT828282	MT827975	MT827850	MT828313
A/duck/Zhejiang/B2039-2/2012(H6N6)	ZJ/B2039	10^6.5^	MT828558	MT828849	MT829182	MT826253	MT828283	MT827976	MT827851	MT828314
A/duck/Zhejiang/B2044-2/2012(H6N6)	ZJ/B2044	10^7.8^	MT828559	MT828850	MT829183	MT826254	MT828284	MT827977	MT827852	MT828315
A/duck/Zhejiang/B1994-2/2012(H6N6)	ZJ/B1994	10^7.3^	MT828560	MT828851	MT829184	MT826255	MT828285	MT827978	MT827853	MT828316
A/duck/Zhejiang/B2028-1/2012(H6N6)	ZJ/B2028	10^6.8^	MT828561	MT828852	MT829185	MT826256	MT828286	MT827979	MT827854	MT828317
A/duck/Guangdong/D1501-1/2014(H6N6)	GD/D1501	10^8.3^	MT828562	MT828853	MT829186	MT826257	MT828287	MT827980	MT827855	MT828318
A/duck/Fujian/D3480-1/2014(H6N6)	FJ/D3480	10^7.8^	MT828563	MT828854	MT829187	MT826258	MT828288	MT827981	MT827856	MT828319
A/duck/Jiangsu/E1201-2/2015(H6N6)	JS/E1201	10^8.5^	MT828564	MT828855	MT829188	MT826259	MT828289	MT827982	MT827857	MT828320
A/duck/Guangdong/E3415-2/2015(H6N6)	GD/E3415	10^7.5^	MT828565	MT828856	MT829189	MT826260	MT828290	MT827983	MT827858	MT828321
A/duck/Guangdong/E3503-2/2015(H6N2)	GD/E3503	10^8.8^	MT828566	MT828857	MT829190	MT826261	MT828291	MT827984	MT827859	MT828322
A/duck/Guangdong/E3724-1/2015(H6N6)	GD/E3724	10^7.8^	MT828567	MT828858	MT829191	MT826262	MT828292	MT827985	MT827860	MT828323
A/duck/Guangdong/E3742-2/2015(H6N2)	GD/E3742	10^8.5^	MT828568	MT828859	MT829192	MT826263	MT828293	MT827986	MT827861	MT828324
A/duck/Guangdong/E3780-1/2015(H6N2)	GD/E3780	10^8.8^	MT828569	MT828860	MT829193	MT826264	MT828294	MT827987	MT827862	MT828325
A/duck/Guangdong/E3798-1/2015(H6N6)	GD/E3798	10^6.5^	MT828570	MT828861	MT829194	MT826265	MT828295	MT827988	MT827863	MT828326
A/duck/Guangdong/F1473-2/2016(H6N2)	GD/F1473	10^8.8^	MT828571	MT828862	MT829195	MT826266	MT828296	MT827989	MT827864	MT828327
A/duck/Jiangsu/F336-2/2016(H6N6)	JS/F336	10^8.3^	MT828572	MT828863	MT829196	MT826267	MT828297	MT827990	MT827865	MT828328
A/duck/Guangdong/F3891-1/2016(H6N2)	GD/F3891	10^8.5^	MT828573	MT828864	MT829197	MT826268	MT828298	MT827991	MT827866	MT828329
A/duck/Jiangsu/G91-1/2017(H6N6)	JS/G91	10^6.3^	MT828574	MT828865	MT829198	MT826269	MT828299	MT827992	MT827867	MT828330
A/duck/Jiangsu/G93-1/2017(H6N6)	JS/G93	10^7.5^	MT828575	MT828866	MT829199	MT826270	MT828300	MT827993	MT827868	MT828331

^a^Viruses whose PB2, PB, PA, HA, NP, NA, M and NS genes were sequenced in the present study.

### Molecular characterization

According to sequence analysis, all of the H6 isolates shared the same amino acids (PQIETR/GLF) with single basic amino acid at the cleavage site between HA1 and HA2, which displayed a low pathogenic feature^32^. The receptor-binding sites in HA protein possessed the amino acid residues Q226 and G228 (H3 HA numbering^33^, which is used throughout the manuscript), suggesting that all H6 isolates would have a higher affinity to α-2,3-linked sialic acid which is predominant in the upper respiratory tract of avian species^34^. Several mutations were found in the receptor-binding area in the HA proteins and listed in Table 2, including the amino acids from 169 to 171 which related to a potential glycosylation site, and the amino acid at 190 which was found to determine the binding capability of H9N2 viruses to lung epithelial cells of mouse and human^35^. The amino acid changes of A138S and P186T/I were appeared in ZJ/B1994/H6N6, FJ/D3480/H6N6and JS/F336/H6N6.

**Table 2:** Molecular characteristics of H6 viruses.

Viruses	Key positions in HA^a^													Amino acid Deletion in NA(Position)	M2	PB2		NS1	PA
	137	138	169–171	186	187	190	192	226–228			156	263	380		31	627	701	92	38
HN/A729	S	A	NNT	P	V	E	**E**	Q	R	G	K	K	K	No	S	E	D	D	I
GD/1268	S	A	NNT	P	**N**	**A**	N	Q	R	G	K	**T**	K	No	S	E	D	D	I
GD/1127	S	A	NNT	P	**N**	**A**	N	Q	R	G	K	**T**	K	No	S	E	D	D	I
HN/2282	S	A	NT**G**	P	V	E	**E**	Q	R	G	K	K	K	No	S	E	D	D	I
ZJ/B2039	S	A	**R**NT	P	**D**	E	N	Q	R	G	K	K	K	No	S	E	D	D	I
ZJ/B2044	S	A	**R**NT	P	**D**	E	N	Q	R	G	K	K	K	No	S	E	D	D	I
ZJ/B1994	S	S	**R**NT	**T**	**D**	E	N	Q	R	G	K	K	K	**(58–68)**	S	E	D	D	I
ZJ/B2028	S	A	**R**NT	P	**D**	E	N	Q	R	G	K	K	K	No	S	E	D	D	I
GD/D1501	S	A	**R**NT	P	**D**	E	N	Q	R	G	K	K	K	No	S	E	D	D	I
FJ/D3480	S	S	**R**NT	**I**	**D**	E	N	Q	R	G	K	K	K	**(58–68)**	S	E	D	D	I
JS/E1201	S	A	NNT	P	V	E	**E**	Q	R	G	K	K	K	No	S	E	D	D	I
GD/E3415	S	A	TNT	P	**D**	E	N	Q	R	G	K	K	K	No	S	E	D	D	I
GD/E3503	S	A	NNT	P	**N**	**A**	N	Q	R	G	K	**T**	K	No	**N**	E	D	D	I
GD/E3724	S	A	**T**NT	P	**D**	E	N	Q	R	G	K	K	K	No	S	E	D	D	I
GD/E3742	S	A	**Y**NT	P	**N**	**A**	N	Q	R	G	K	**T**	K	No	S	E	D	D	I
GD/E3780	S	A	NNT	P	**N**	A	**D**	Q	R	G	K	**T**	K	No	S	E	D	D	I
GD/E3798	S	A	NNT	P	V	E	**E**	Q	R	G	K	K	K	No	S	E	D	D	I
GD/F1473	S	A	NNT	P	V	E	**E**	Q	R	G	K	K	K	No	S	E	D	D	I
JS/F336	S	S	**R**NT	**I**	**D**	E	N	Q	R	G	K	K	K	**(58–68)**	S	E	D	D	I
GD/F3891	S	A	NNT	P	**N**	**A**	N	Q	R	G	K	**T**	K	No	**N**	E	D	D	I
JS/G91	S	A	**T**NN	P	**D**	E	N	Q	R	G	K	K	K	No	S	E	D	D	I
JS/G93	S	A	**T**NN	P	**D**	E	N	Q	R	G	K	K	K	No	S	E	D	D	I

Residues in bold indicate differences from the consensus alignment.

^a^H3 numbers were used throughout.

Those 22 viruses contained 119E, 274H and 294 N on the NA proteins (N2 numbering^36^), which suggested that all viruses are sensitive to oseltamivir^37^. There were 11 amino acids deletion in the stalk region of three H6N6 isolates (ZJ/B1994/H6N6, FJ/D3480/H6N6 and JS/F336/H6N6), which might be associated with increased virulence in mammals^38^. It is notable that S31N substitutions in the M2 protein, which are associated with amantadine resistance of influenza virus were observed in GD/E3503/H6N2 and GD/F3891/H6N2 viruses^39^. Amino acid residues were E and D at positions 627 and 701 of the PB2 protein in all those H6 isolates, which have been associated with the pathogenicity of avian influenza viruses in mammals^40^. Previous reports showed that the substitution D92E in the NS1 protein associated with reducing its phosphorylation and increasing the virus resistance to interferon^41^, but this substitution was not detected in any of the isolates. In addition, no amino acid changes associated with increased virulence in mammals were detected in the PA or PB1 proteins.

### Phylogenetic analysis of HA and NA genes

**Figure 1:**
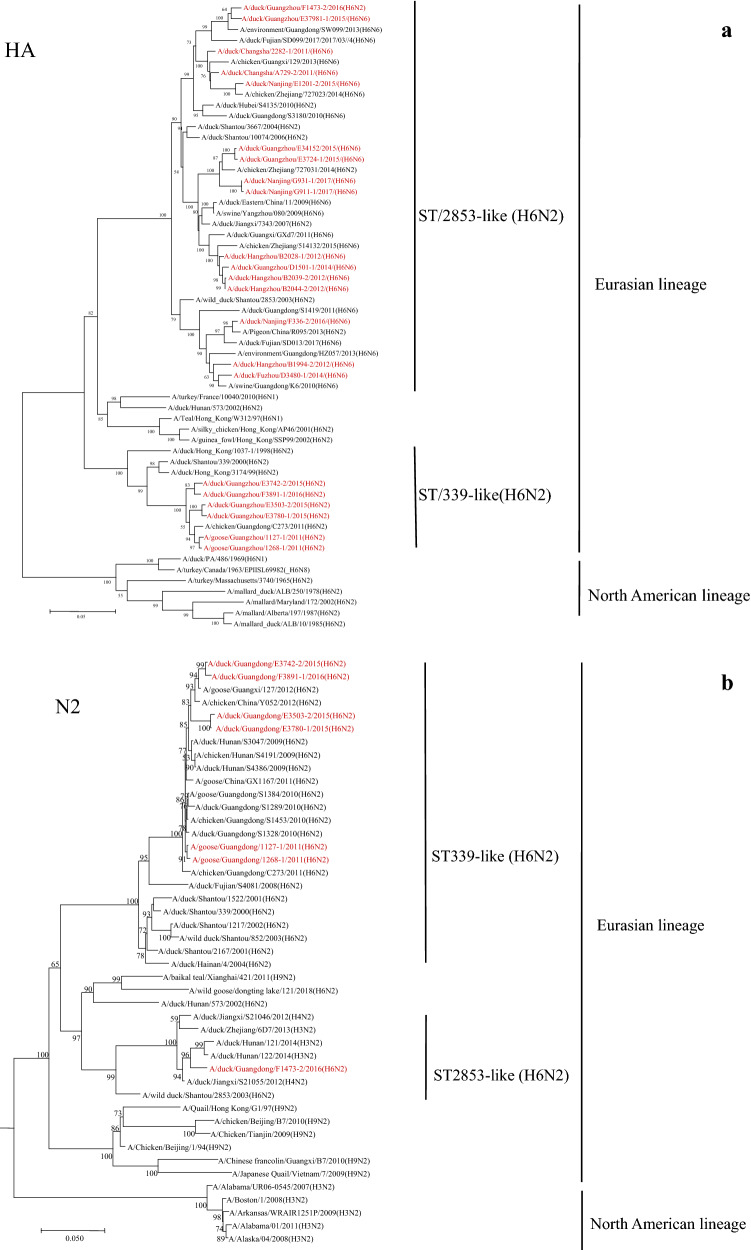

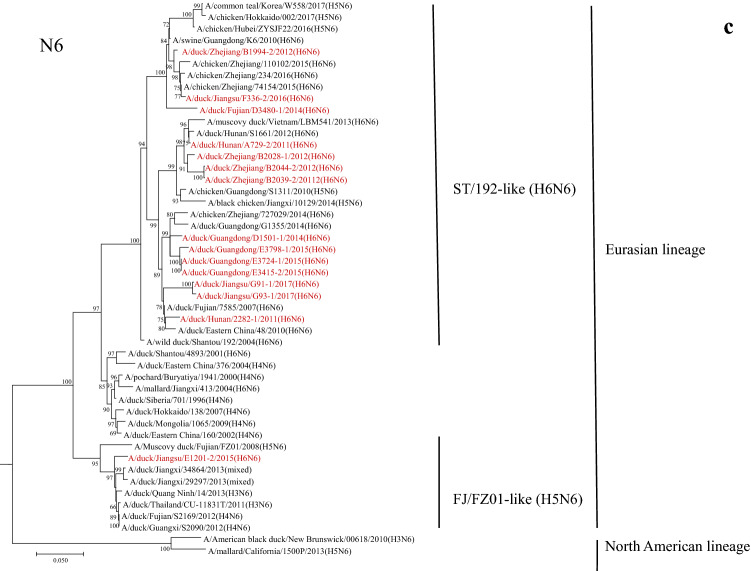
Phylogenetic trees of H6 AIVs isolated from 2011 to 2017 in Southern China. The phylogenetic trees of the H6 HA (**a**), N2 NA (**b**) and N6 NA (**c**) genes were generated by using the neighbor-joining method using MEGA 6.0. The genomic sequences of the viruses listed in black were downloaded from available databases; the viruses listed in red were evaluated in this study. The scale bar represents the distance between sequence pairs, and horizontal distances are proportional to genetic distance.

The nucleotide and amino acid similarity of the HA from 22 isolates in this study were 82.0% to 99.9% and 84.0% to 99.8%, respectively, as shown in Table 3. The phylogenetic tree of HA gene (Fig. 1a) indicated that all of the H6 isolates were divided into two clades under Eurasian lineage. All the H6N2 isolates were clustered in A/duck/Shantou/339/2000(H6N2)-like (ST/339-like) virus clade except GD/F1473/H6N2. Together with all of the H6N6 viruses, GD/F1473/H6N2 was derived from A/wild duck/Shantou/2853/2003(H6N2)-like (ST/2853-like) virus clade. Additionally, 3 H6N6 strains (ZJ/B1994/H6N6, FJ/D3480/H6N6 and JS/F336/H6N6) belonged to A/swine/Guangdong/K6/2010(H6N6)-like (GD/K6-like) subclade.

**Table 3:** Nucleotide and amino acid identity of the HA genes among the twenty-two H6 subtype isolates from Southern China, 2011–2017.

Virus	Nucleotide identity (%)																					
	HN/A729	GD/1268	GD/1127	HN/2282	ZJ/B2039	ZJ/B2044	ZJ/B1994	ZJ/B2028	GD/D1501	FJ/D3480	JS/E1201	GD/E3415	GD/E3503	GD/E3724	GD/E3742	GD/E3780	GD/E3798	GD/F1473	JS/F336	GD/F3891	JS/G91	JS/G93
HN/A729		83.1	82.9	97.8	93.2	94.1	92	94.2	94	92.1	96.9	93.1	82.9	93	83	83	95.1	94.9	91.7	82.9	92.4	92.5
GD/1268	86.9		99.5	82.6	82.2	82.9	82.4	83.1	82.9	82.1	82.8	82.4	98.3	82.3	97.9	97.9	82.4	82.7	82.1	97	82.1	82.2
GD/1127	86.9	99.3		82.4	82	82.7	82.3	83	82.7	82	82.7	82.3	98.1	82.2	97.8	97.8	82.3	82.5	82	96.9	82.1	82.2
HN/2282	97.7	85.7	85.7		94.3	93.7	91.2	93.8	93.5	91.2	95.6	92.5	82.5	92.4	82.5	82.6	94.5	94.4	90.8	82.5	91.9	92.1
ZJ/B2039	94	85.2	85.2	95.1		99.1	91.3	98.4	98.4	91.2	91.3	94.1	82	94.1	82.2	82	91.5	91.7	90.7	82.4	93.5	93.7
ZJ/B2044	95.4	86.2	86.2	94.4	98.6		92.2	99.2	99.4	92.1	92.2	95	82.6	94.9	82.9	82.5	92.4	92.5	91.6	83.1	94.4	94.5
ZJ/B1994	92.4	85.7	86.1	91.2	92.9	94.4		92.3	91.9	97.2	90.8	91.5	82.2	91.5	82.6	82.1	90.5	90.4	97.6	83	90.9	91
ZJ/B2028	95.6	86.4	86.4	94.5	98.4	99.8	94.5		98.8	92.2	92.4	95	82.8	94.9	83	82.8	92.5	92.5	91.7	83.1	94.6	94.7
GD/D1501	94.9	85.9	85.9	94	97.7	99.1	93.5	98.9		92.1	92	94.8	82.6	94.8	82.9	82.5	92.2	92.2	91.3	83	94.4	94.5
FJ/D3480	92.4	85.7	86.1	91.2	92.6	94	97.9	93.8	93.5		90.8	91.7	81.9	91.7	82.3	81.8	90.7	90.5	96.3	82.5	91	91.1
JS/E1201	98.6	87.1	87.1	96.6	93.1	94.5	91.9	94.7	94	91.9		91.5	82.6	91.4	82.8	82.7	93.5	93.4	90.2	82.9	91.4	91.5
GD/E3415	94	85	85	92.9	95.4	96.8	92.4	97	95.9	91.7	93.5		82.5	99.7	82.8	82.4	91.9	92.2	91.2	83.1	96.9	97
GD/E3503	86.2	97.9	98.2	85	84.7	85.7	85.4	85.9	85.2	85.4	86.4	84.7		82.4	98.3	99.4	82	82.3	82.1	97.5	82.1	82.2
GD/E3724	93.7	84.7	84.7	92.6	95.1	96.5	92.1	96.6	95.6	91.4	93.1	99.6	84.3		82.8	82.4	91.8	91.9	91.2	83.1	96.8	96.9
GD/E3742	86.4	97.9	98.2	85.2	85.2	86.2	86.2	86.4	85.9	86.2	86.9	85.2	98.6	84.8		97.9	82.5	82.7	82.4	98.9	82.5	82.6
GD/E3780	86.1	97.5	97.9	84.8	84.3	85.4	85	85.5	85.2	85	86.2	84.3	98.9	84	98.2		82	82.4	82	97.2	82.1	82.2
GD/E3798	97.2	86.4	86.4	96.1	93.1	94.4	91.9	94.5	93.8	91.9	96.1	93.5	85.7	93.1	85.9	85.5		98.4	90	82.5	91.5	91.6
GD/F1473	96.8	86.2	86.2	95.8	92.9	94.2	91.7	94.4	93.7	91.7	95.8	93.1	85.5	92.8	85.7	85.4	98.8		90.2	82.7	91.7	91.8
JS/F336	92.2	85.9	86.2	91	93.3	94.7	98.9	94.9	93.8	98.2	91.7	92.9	85.9	92.6	86.8	85.5	91.7	91.5		82.8	90.3	90.4
GD/F3891	86.2	97.5	97.9	85.4	85.4	86.4	86.4	86.6	86.1	86.4	86.8	85	98.2	84.7	98.9	97.9	86.4	86.4	86.9		82.8	82.9
JS/G91	92.6	84.5	84.5	91.5	94	95.4	91.5	95.6	94.9	91.2	92.8	96.8	84.1	96.5	84.7	83.8	92.6	92.4	92.1	84.8		99.9
JS/G93	92.9	84.8	84.8	91.9	94.4	95.8	91.9	95.9	95.2	91.5	93.1	97.2	84.5	96.8	85	84.1	92.9	92.8	92.4	85.2	99.6	
	Amino acid identity (%)

The NA genes of 7 H6N2 isolates separated into two clades (ST/339-like and ST/2853-like clades). Only GD/F1473 clustered in the ST/2853-like clade, in which H3N2, H4N2, and H6N2 viruses were clustered. The other H6N2 viruses fell into ST/339-likeclade, in which the viruses were isolated from ducks, geese and chickens (Fig. 1b). The NA genes of 15 H6N6 isolates were divided into two clades as well. The majority of those viruses were clustered together with the clade of ST/192-like viruses. Only the JS/E1201/H6N6 strain clustered on the clade of A/Muscovy duck/Fujian/FZ01/2008(H5N6)-like (FJ/FZ01-like) virus (Fig. 1c).

### Phylogenetic analysis of the internal genes

Phylogenetic analysis of the six internal genes showed that all those 22 H6 AIVs clustered in the Eurasian lineage. The PB1, PB2, PA and NS genes were divided into two clades, and the NP and M genes were fell into 3 clades (Fig. S1-6). Phylogenetic tree of PB1 genes showed that nine isolates were clustered in the ST339-like clade, in which the viruses were isolated from different birds including chicken, duck, goose and wild bird. The other thirteen isolates were closely related to A/duck/Guangdong/S3180/2010(H6N6) (GD/S3180) (Fig. S1). In the phylogenetic trees of the PB2 and NS genes, only GD/F1473/H6N2 located in a separated clade (Fig. S2 and S6). PB2 gene of GD/F1473/H6N2 was related closely to that of the early H5N1 AIV isolate A/goose/Guangdong/1/96 in the BJ/BJ/1/94-likeclade. The NS gene of GD/F1473/H6N2 was closely related to that of H3N2, H3N8, and H6N2 AIVs isolated from different waterfowls. As for the PA genes, all of the H6N6 viruses and one H6N2 virus GD/F1473/H6N2 were clustered in the ST339-like clade (Fig. S3). The other 5H6N2 viruses were located in the Gs/GD196-like clade. The NP gene of the virus JS/E1201/H6N6 was clustered in a separated A/duck/Hunan/573/2002(H6N2)-like (HN/573-like) clade, while other isolates were divided into ST339-likeand A/duck/Mongolia/54/2001(H5N2)-like (Mongolia/54-like) virus groups (Fig. S4). On the NP tree, most of the H6N6 and GD/F1473/H6N2were related to a H5N2 virus A/duck/Mongolia/54/2001. In the ST339-like clade, the NP genes of 6H6N2 isolates clustered into a A/chicken/Guangdong/C273/2011 related sub-group, while 3 H6N6 viruses clustered into the A/swine/Guangdong/K6/2010 related sub-group. In the phylogenetic tree of M gene, all the viruses were distributed in ST/339-like, China/G1291-like and ST/2853-like clades (Fig. S5). The M genes of 6 H6N2 viruses located in China/G1291-like, 3 H6N6 viruses located in ST/2853-like clade, and the most of H6N6 viruses clustered together with a H6N2 isolate GD/F1473/H6N2 on ST/339-like clade.

**Figure 2:**
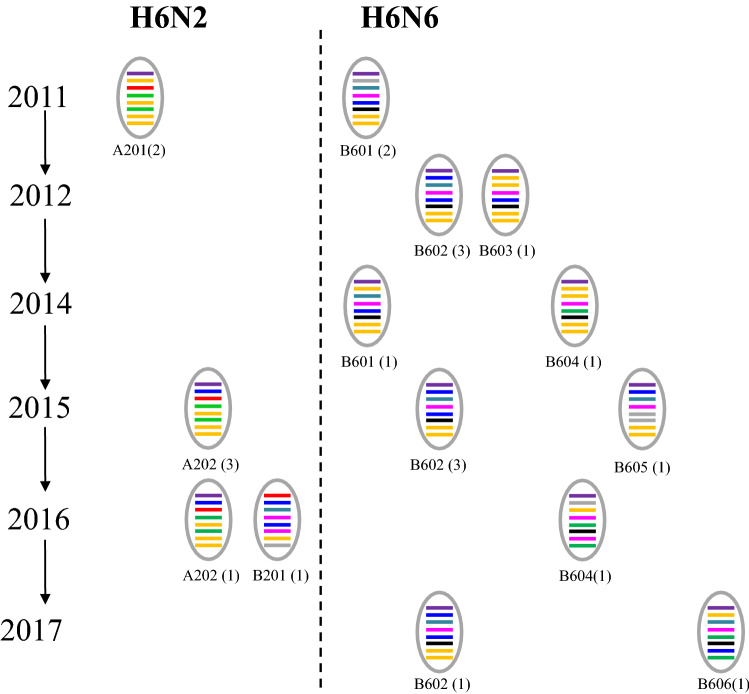
Genotypes of H6 AIVs isolated from 2011 to 2017 in Southern China. The genotypes of H6 influenza viruses were determined by the clades of each of their gene segments on the phylogenetic trees.

According to the phylogenetic analysis of the whole genomes of the AIVs isolated from 2011 to 2017, the genotypes of H6 viruses were classified. Based on the HA genes, the viruses were divided into A and B genotypes corresponding to ST/339-like and ST/2853-like clades. H6N2 and H6N6 viruses were divided into A2, B2, A6 and B6 accordingly. Based on the different clades of NA and other 6 internal genes, the genotypes were named according to the chronological order of virus isolation in this study (Fig. 2). The genetic reassortment of H6 AIVs occurred frequently from 2011 to 2017, and resulted in multiple genotypes. The genotype of B602 was detected in 2012, 2015 and 2017, suggesting that the viruses of this genotype have elevated adaptation and replication efficiency in their natural reservoirs.

### Pathogenicity in mice

**Figure 3:**
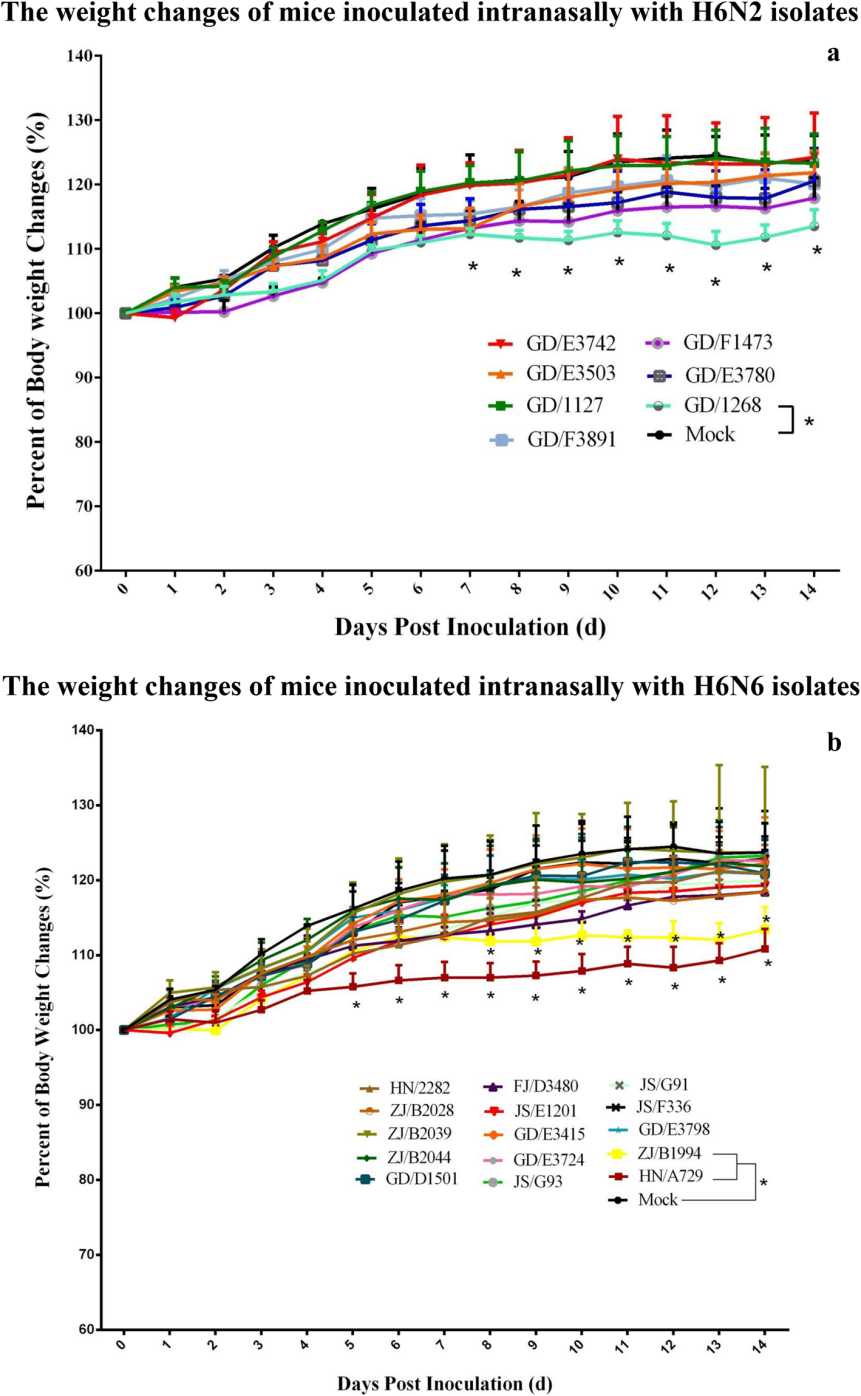
Bodyweight changes of BALB/c mice infected with the H6 AIVs. 4-week-old female BALB/c mice were inoculated i.n. with 10^6.0^EID_50_ of virus in a volume of 30.0 μl. The body weight of 5 mice were measured daily until 14 dpi. Weight changes of the BALB/c mice infected with H6N2 (Fig. 3a) and H6N6 (Fig. 3b) were shown respectively. The data were graphed using the Prism 6.0 software (Vision 6.0, GraphPad Prism). Comparisons of the weight changes between two groups were determined by nonparametric t-tests using GraphPad. (*P < 0.05, **P < 0.01, ***P < 0.001).

To evaluate the pathogenicity in mice, groups of white mice were inoculated i.n. with each H6 isolates (10^6.0^ EID_50_/mice). All of the mice used for observation survived till 14 dpi. None of H6N2 viruses, except GD/1268/H6N2, caused significant body weight loss (P < 0.05) in mice compared with the control group (Fig. 3). As for H6N6 viruses, only HN/A729/H6N6 and ZJ/B1994/H6N6 caused significant growth retard in mice, while the clinical signs caused by other viruses were not obvious.

All of the H6H2 isolates were able to replicate efficiently in mouse lung and nasal turbinate without prior adaptation. The mean virus titers were ranged from 10^3.4^ to 10^6.0^ EID_50_/ml in the lungs and from 10^1.8^ to10^4.8^ EID_50_/ml in the nasal turbinates (Table 4). Six out of seven H6N2 isolates were detectable in the heart of partial or all the three inoculated mice. Specially, two H6N2 viruses were found in the spleen of one out of three inoculated mice, and one of those viruses GD/E3503/H6N2 caused systemic infection in one inoculated mouse. In addition, the virus was recovered from liver and brain. The GD/1268/H6N2 and GD/1127/H6N2 viruses of A201 genotype were detectable in the lungs, nasal turbinates and hearts consistently, while the 4 isolates of A202 genotype showed diversity in the tissue tropisms (Table 4).

**Table 4:** Replication of H6 viruses in mice.

Virus	Genotype	Subtype	Virus titers(lgEID_50_/ml)					
			Lung	Nasal turbinate	Heart	Liver	Spleen	Brain
GD/1268	A201	H6N2	3.42 ± 0.14 ^a^	4.08 ± 0.72	1.25(1/3)^b^	/^c^	/	/
GD/1127	A201	H6N2	4.25 ± 0.66	4.50 ± 0.25	0.98/1.25(2/3)	/	/	/
GD/E3503	A202	H6N2	6.00 ± 0.43	3.83 ± 1.53	2.75 ± 1.32	0.98(1/3)	0.98(1/3)	2.5(1/3)
GD/E3742	A202	H6N2	5.08 ± 0.52	3.75 ± 1.00	1.75/2.25(2/3)	/	/	/
GD/E3780	A202	H6N2	4.75 ± 0.43	4.33 ± 0.14	2.00 ± 0.66	/	/	/
GD/F3891	A202	H6N2	3.75 ± 0.43	4.75 ± 1.09	/	/	1.25(1/3)	/
GD/F1473	B201	H6N2	4.33 ± 0.14	1.75 ± 0.87	1.5/2.5(2/3)	/	/	/
HN/A729	B601	H6N6	2.08 ± 0.72	3.5(1/3)	1.5(1/3)	/	/	/
HN/2282	B601	H6N6	5.50 ± 0.00	1.25(1/3)	1.50(1/3)	2.25(1/3)	/	/
GD/D1501	B601	H6N6	4.42 ± 0.76	0.98(1/3)	3.5(1/3)	/	/	0.98(1/3)
ZJ/B2039	B602	H6N6	/	/	/	/	/	/
ZJ/B2044	B602	H6N6	3.58 ± 2.08	/	1.5/1.5(2/3)	/	/	/
ZJ/B2028	B602	H6N6	5.08 ± 1.89	0.98/1.25(2/3)	2.58 ± 0.76	/	1.5(1/3)	/
GD/E3415	B602	H6N6	4.58 ± 0.88	1.07 ± 0.16	4.25(1/3)	/	/	/
GD/E3724	B602	H6N6	3.25 ± 0.87	1.25(1/3)	1.5/2.75(2/3)	/	/	/
GD/E3798	B602	H6N6	1.25/0.98(2/3)	0.98/0.98(2/3)	/	/	/	/
JS/G93	B602	H6N6	5.33 ± 0.14	2/3(1.25/1.25)	1.07 ± 0.16	/	/	/
ZJ/B1994	B603	H6N6	5.00 ± 0.66	0.98(1/3)	2.50 ± 0.25	1.75(1/3)	/	0.98(1/3)
FJ/D3480	B604	H6N6	6.25 ± 0.00	2.50 ± 0.75	2.00 ± 0.66	/	1.25(1/3)	/
JS/F336	B604	H6N6	4.00 ± 1.39	1.75/1.25(2/3)	1.74 ± 1.31	1.5(1/3)	/	0.98(1/3)
JS/E1201	B605	H6N6	2.57 ± 2.75	0.98(1/3)	2.25(1/3)	0.98(1/3)	/	/
JS/G91	B606	H6N6	/	0.98(1/3)	/	/	/	/

^a^Virus was detected in all of three mice, the virus titers were shown as mean ± SEM.

^b^Virus was detected in part of three mice, the virus titers were shown respectively.

^c^Virus was not detected in any of three mice.

The H6N6 viruses, except ZJ/B2039/H6N6 and JS/G91/H6N6, replicated in the lungs of inoculated mice. ZJ/B2039/H6N6 has not been detected in any of the other tested tissues, and lower titers of JS/G91/H6N6 were detected only in the nasal turbinate of one out of three mice (Table 4), suggesting the limited replication of those 2 viruses in mice. In addition, the replication of GD/E3798/H6N6 was also limited in mice and lower virus titers were detected in the lungs and nasal turbinates from two out of three mice. Besides those 3 viruses, the other H6N6 isolates replicated well in the lungs and the virus titers ranged from 10^2.1^ to 10^6.3^ EID_50_/ml. Specially, the average virus titers of 5 isolates reached a high level of over 10^5*.*0^ EID_50_/ml, which suggested those viruses replicated efficiently in the lungs of inoculated mice. In the nasal turbinates, only GD/E3415/H6N6 and FJ/D3480/H6N6 could be detected in all of the three mice, and the average virus titers were 10^1.1^ and 10^2.5^ EID_50_/ml, respectively. Except ZJ/B2039/H6N6 and ZJ/B2044/H6N6 which were not recovered from the nasal turbinates of all three mice, the remaining H6N6 viruses were detected in some inoculated mice. All of the H6N6 isolates were detectable in the hearts of some to all of the three inoculated mice, except ZJ/B2039/H6N6, GD/E3798/H6N6 and JS/G91/H6N6. Lower viral titers of some H6N6 viruses were detected in livers, spleens and brains in some inoculated mice, suggesting that those viruses replicate in mice (Table 4).

### Pathogenicity and transmission in chickens

To evaluate the virulence of the H6 isolates in chickens, groups of six 4-week-old SPF chickens were inoculated intranasally with 10^6.0^ EID_50_ of each H6 isolate. No clinical sign was observed in the inoculated chickens. We further examined the replication of the H6 viruses by detecting the viruses in different tissue and swabs of inoculated chickens. The GD/1127/H6N2 and GD/E3503/H6N2 viruses were detected in the trachea of one inoculated chicken, respectively. FJ/D3480/H6N6 was detected in the duodenum of one inoculated chicken, while HN/A729/H6N6 and ZJ/B2028/H6N6 were not detected in any tested tissues of 3 inoculated chickens at 3dpi. Only GD/E3503/H6N2 was detected in the oropharyngeal swab from one chicken at 4 dpi. GD/E3503/H6N2 and HN/A729/H6N6 were detected in the cloacal swab from one chicken at 2 dpi and 6 dpi, respectively. None of 3 chickens inoculated with HN/A729/H6N6 showed seropositive to specific H6 antibody, and only some chickens inoculated with the other four viruses were seroconverted. One contact chicken in the GD/E3503/H6N2 group was seroconverted at 14 dpi, while no specific H6 antibody was detected in other contact chickens (Table 5). The results suggested the replication and transmission of H6 viruses with different genotypes were limited in chickens.

**Table 5:** Replication of the H6 viruses in chickens.

Viruses	Genotypes	Virus isolation											Antibody test	
		Trachea	Lungs	Spleen	Kidneys	Duodenum	Oropharyngeal swabs			Cloacal swabs				
							2 dpi	4 dpi	6 dpi	2 dpi	4 dpi	6 dpi	Inoculated	contact
GD/1127	A201	1/3^a^	0/3	0/3	0/3	0/3	0/3	0/3	0/3	0/3	0/3	0/3	2/3^b^	0/3
GD/E3503	A202	1/3	0/3	0/3	0/3	0/3	0/3	1/3	0/3	1/3	0/3	0/3	2/3	1/3
HN/A729	B601	0/3	0/3	0/3	0/3	0/3	0/3	0/3	0/3	0/3	0/3	1/3	0/3	0/3
ZJ/B2028	B602	0/3	0/3	0/3	0/3	0/3	0/3	0/3	0/3	0/3	0/3	0/3	1/3	0/3
FJ/D3480	B604	0/3	0/3	0/3	0/3	1/3	0/3	0/3	0/3	0/3	0/3	0/3	1/3	0/3

^a^The number of virus positive samples/total.

^b^The number of antibody positive samples/total, the HI titers ≥ 4 were considered as antibody positive samples.

## Discussion

Since the late 1990s, H6 AIVs have been circulating in Southern China. The H6 viruses have been become more prevalent over time with an increasing isolation rate of H6N1, H6N2 and H6N6 subtypes^6,15,42^. In this study, we analyzed the molecular evolution and pathogenicity in mice and chickens of H6 AIVs isolated from the LPMs in Southern China from 2011 to 2017, and provided a glimpse of the genetic diversity and pathogenicity of H6 AIVs in mammals and chickens.

Multiple H6 virus genotypes emerged from 2011 to 2017 resulted from the internal gene reassortment between H6 and other subtype viruses. It has been noted that most of the H6N1/N2 viruses isolated from Southern China from 2000 to 2005 were clustered with the G1-like, W312-like or H9N2 Ck/Bei-like lineage based on the PB2 and PB1 genes, and the PA and NP genes of most H6 viruses clustered with H5 and H9 viruses, M and NS genes with G1-like or H9N2 Ck/Bei-like lineage viruses^16,22^. Previous studies showed that the H6 isolate from ducks replicated poorly in chicken trachea and that the viruses recovered were W312-like viruses^43,44^. However, in our study, none of the internal genes were related closely to G1-like or W312-like viruses. These results suggested that the H6 subtype viruses in Southern China were genetically diverse, which might increase the potential for H6 viruses to transmit from ducks to other animals.

Molecular analyses suggested that all of the 22 H6 isolates were low pathogenic AIVs. The receptor-binding sites in the viral HA proteins possess the residues Q226 and G228, similar to those H6 isolates reported previously, which preferentially bind to the α-2,3-linked sialic acid receptors predominant in avian species^45,46^. However, the E190V and N192D substitutions of H6 HA have been associated with interspecies transmission of AIVs from ducks to chickens^47^, but those amino acids were not found in the five H6 AIVs (GD/1127/H6N2, GD/E3503/H6N2, HN/A729/H6N6, ZJ/B2028/H6N6 and FJ/D3480/H6N6) in this study, which would be the most likely reason that the replication of the H6 isolates in chickens was surprisingly limited. The residues 228S, 137N, 186L, A13S, and A193N at HA associated with human receptor-binding preference^45,47^ were not found in H6 isolates in this study. Additionally, almost all H6N6 isolates have the substitution of HA at V187D, the binding affinity might be altered to adapt to mammalian receptors^19^.

None of E119V, H275Y, R293K and N295S substitution in the NA were found, which suggested those H6 isolates are sensitive to neuraminidase inhibitors such as oseltamivir. The deletion of 11 amino acids in the stalk region of the NA of FJ/D3480/H6N6, ZJ/B1994/H6N6and JS/F336/H6N6, which was also found in a swine H6N6 virus, might be associated with the infectivity of H6N6 viruses in mammals through affecting NA activity^48^ and the balance between HA and NA^49^. It is notable that the S31N substitution in the M2 protein, which is associated with amantadine resistance of influenza virus^50^, was found in GD/E3503/H6N2 and GD/F3891/H6N2.

Some of the H6 viruses replicated efficiently on MDCK and A549 cells and in the lungs of mice^17,51^. The direct contact transmission of H6 viruses in guinea pigs was confirmed previously, which suggested the H6 viruses pose a clear threat to mammals^17^. We found that all of the H6H2 isolates were able to replicate efficiently in lung and nasal turbinate without prior adaptation in mice, but the replication ability of H6N6 varies. Even with the same genotype, different H6N6 viruses showed distinct replication abilities in mice, suggesting some amino acid mutations affected the replication of the viruses. Lower titers of some H6 viruses were detected in livers, spleens and brains in a part of inoculated mice, which suggested those viruses posed a potential to adapt and caused a systemic infection in mammals.

Five H6 AIVs selected from different genotypes caused no clinical signs in any of the inoculated chickens. The replication and transmission of H6N2 and H6N6 viruses were limited in SFP chicken in this study, although the pathological changes caused by H6N2 in chickens were reported in California^13^. Previous study showed that H6N2 isolates caused seroconversion in infected chickens, but no virus was recovered from the tissue samples, which suggested that the H6N2 strains replicated poorly and were nonpathogenic to chickens^52^. Our findings showed that the five H6 strains selected from different genotypes caused no clinical signs in any of the inoculated chickens, but the serological test results suggested that chickens in four groups have been infected despite limited recovery of inoculated viruses from the tissue samples. The replication and transmission of H6N2 and H6N6 viruses were limited in SFP chicken in this study.

Overall, our study suggested that the H6 AIVs circulating in South China are genetically diverse and pose potential threat to mammals, and continual surveillance of H6 viruses is necessary in China.

## Supplementary information

Supplementary information.
