# Supplementary material for: Evolution and pathogenicity of H6 avian influenza viruses isolated from Southern China during 2011 to 2017 in mice and chickens
Source: Sci Rep. 2020 Nov 25;10:20583. doi: 10.1038/s41598-020-76541-0 (PMC7689535; doi:10.1038/s41598-020-76541-0)
Supplement: Supplementary file 1 — Supplementary information. [file 41598_2020_76541_MOESM1_ESM.pdf]

supplementary information

Evolution and pathogenicity of H6 avian influenza viruses isolated from Southern China during 2011 to 2017 in mice and chickenst

Weishan Lin[1,2 §], Hongrui Cui[2 §], Qiaoyang Teng[2], Luzhao Li[2], Ying shi[2], Xuesong Li[2], Jianmei Yang[2], Qinfang Liu[2], Junliang Deng[1]*,Zejun Li[2]*

[1] College of Veterinary Medicine, Sichuan Agricultural University, Chengdu, China.

[2] Shanghai Veterinary Research Institute, Chinese Academy of Agricultural Sciences, Shanghai, People's Republic of China.

[§] These authors contributed equally

*Correspondence: ZejunLi, lizejun@shvri.ac.cn, JunliangDeng, dengjl213@ 126.com

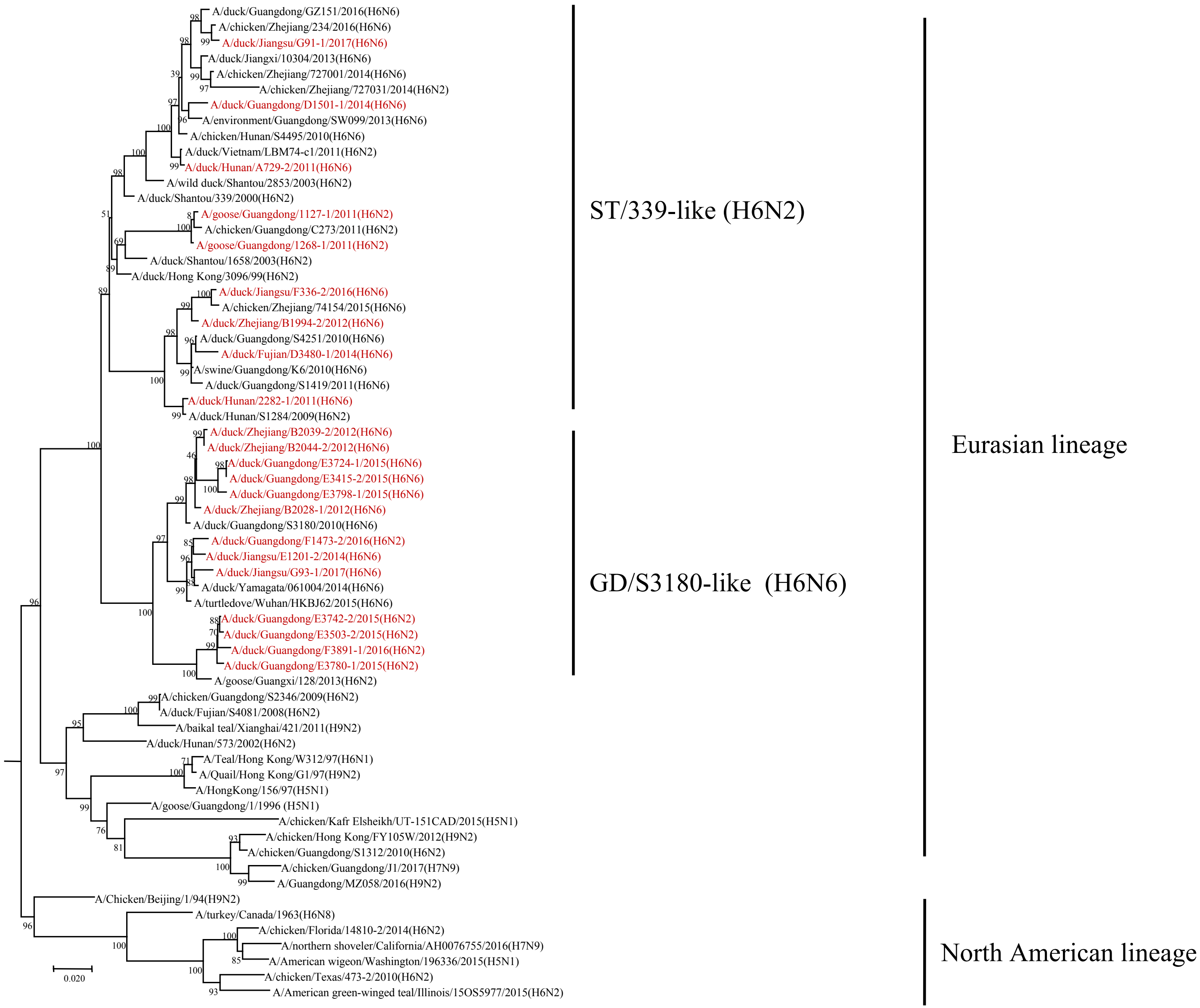

Fig. S1 Phylogenetic trees of PB1 of H6 AIVs. The phylogenetic tree was generated by the neighbor-joining method using MEGA 6.0. The viruses listed in black were downloaded from available databases; the viruses listed in red were evaluated in this study. The scale bar represents the distance between sequence pairs, and horizontal distances are proportional to genetic distance.

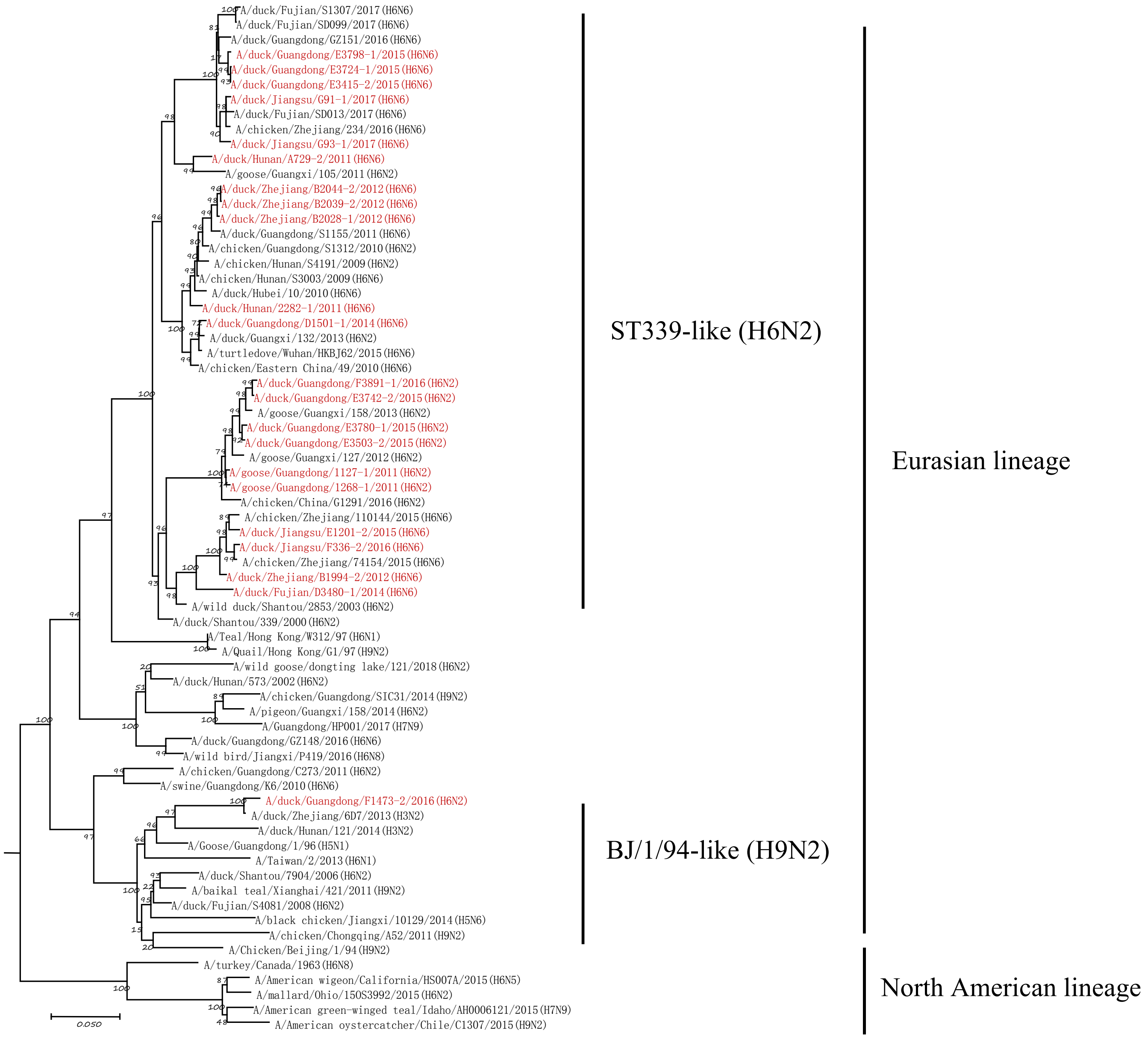

Fig. S2 Phylogenetic trees of PB2 of H6 AIVs. The phylogenetic tree was generated by the neighbor-joining method using MEGA 6.0. The viruses listed in black were downloaded from available databases; the viruses listed in red were evaluated in this study. The scale bar represents the distance between sequence pairs, and horizontal distances are proportional to genetic distance.

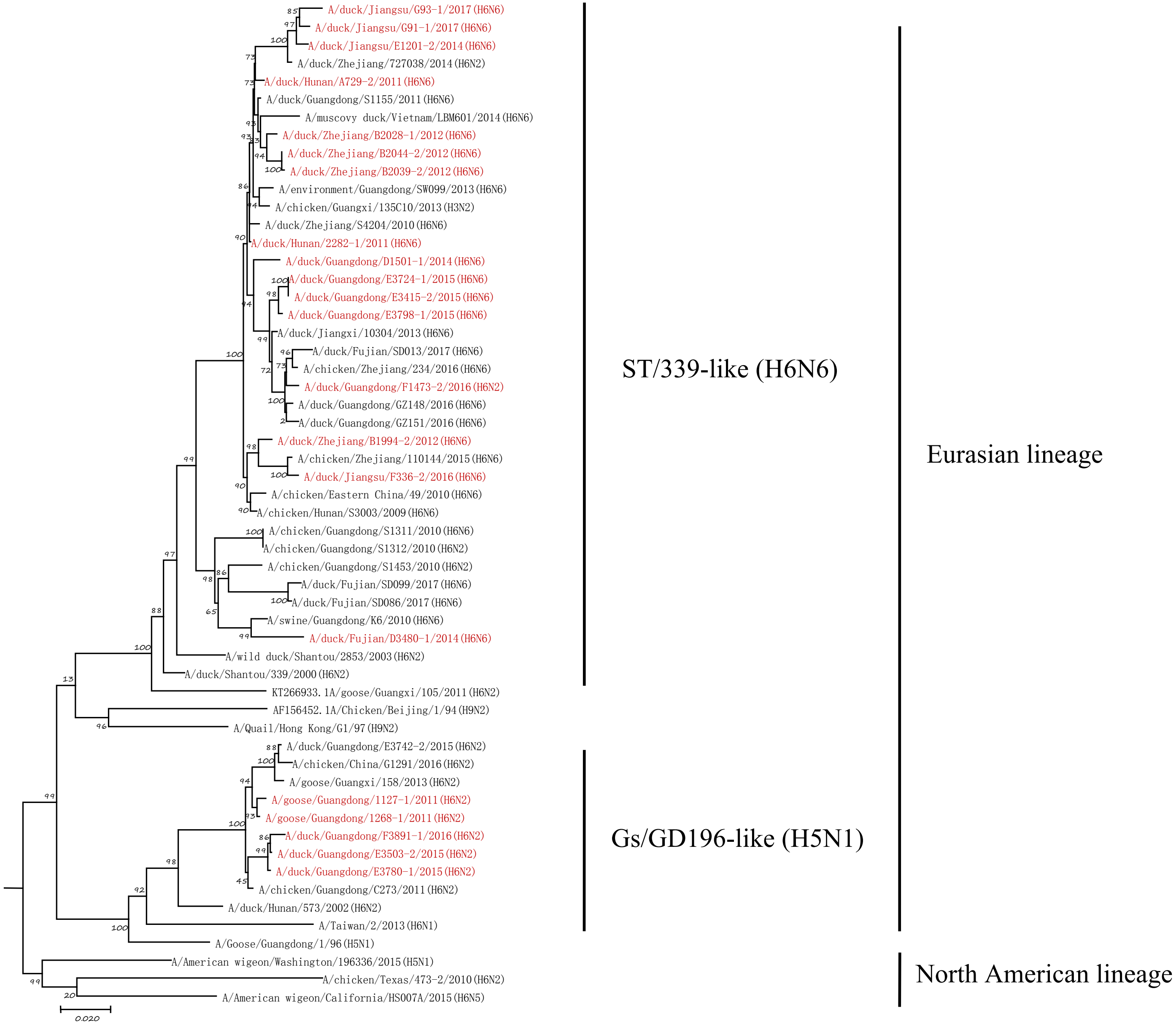

Fig. S3 Phylogenetic trees of PA of H6 AIVs. The phylogenetic tree was generated by the neighbor-joining method using MEGA 6.0. The viruses listed in black were downloaded from available databases; the viruses listed in red were evaluated in this study. The scale bar represents the distance between sequence pairs, and horizontal distances are proportional to genetic distance.

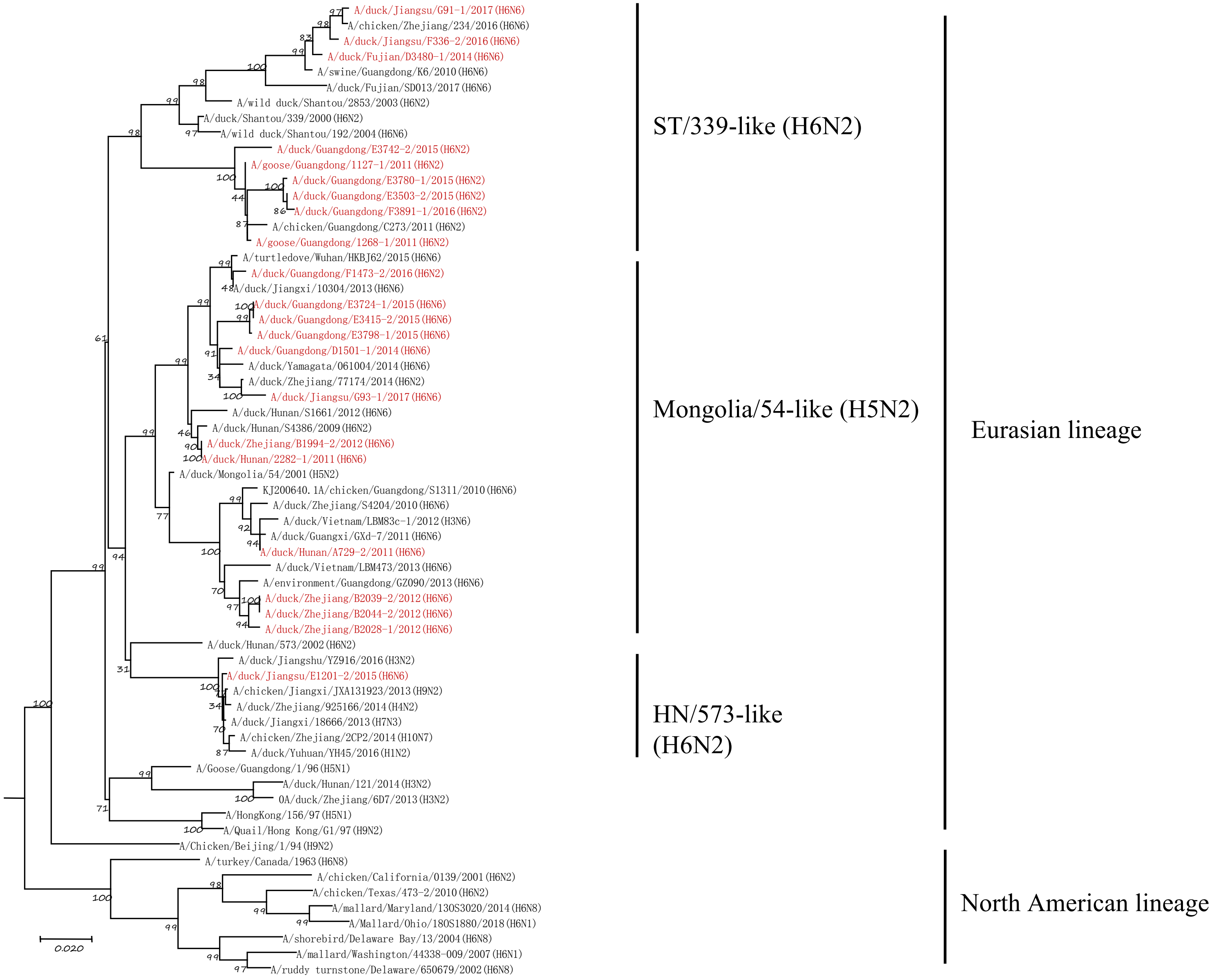

Fig. S4 Phylogenetic trees of NP of H6 AIVs. The phylogenetic tree was generated by the neighbor-joining method using MEGA 6.0. The viruses listed in black were downloaded from available databases; the viruses listed in red were evaluated in this study. The scale bar represents the distance between sequence pairs, and horizontal distances are proportional to genetic distance.

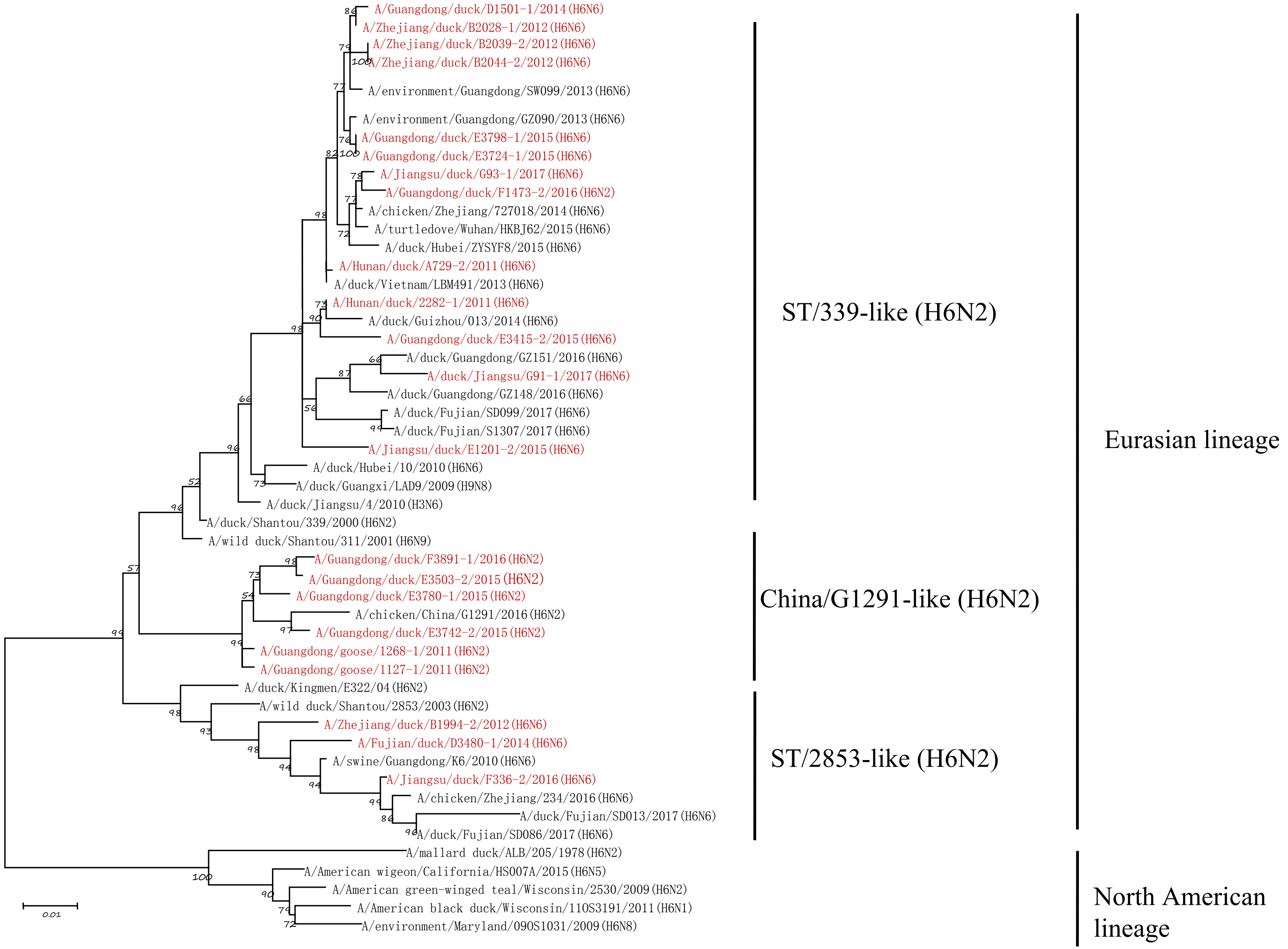

Fig. S5 Phylogenetic trees of M of H6 AIVs. The phylogenetic tree was generated by the neighbor-joining method using MEGA 6.0. The viruses listed in black were downloaded from available databases; the viruses listed in red were evaluated in this study. The scale bar represents the distance between sequence pairs, and horizontal distances are proportional to genetic distance.

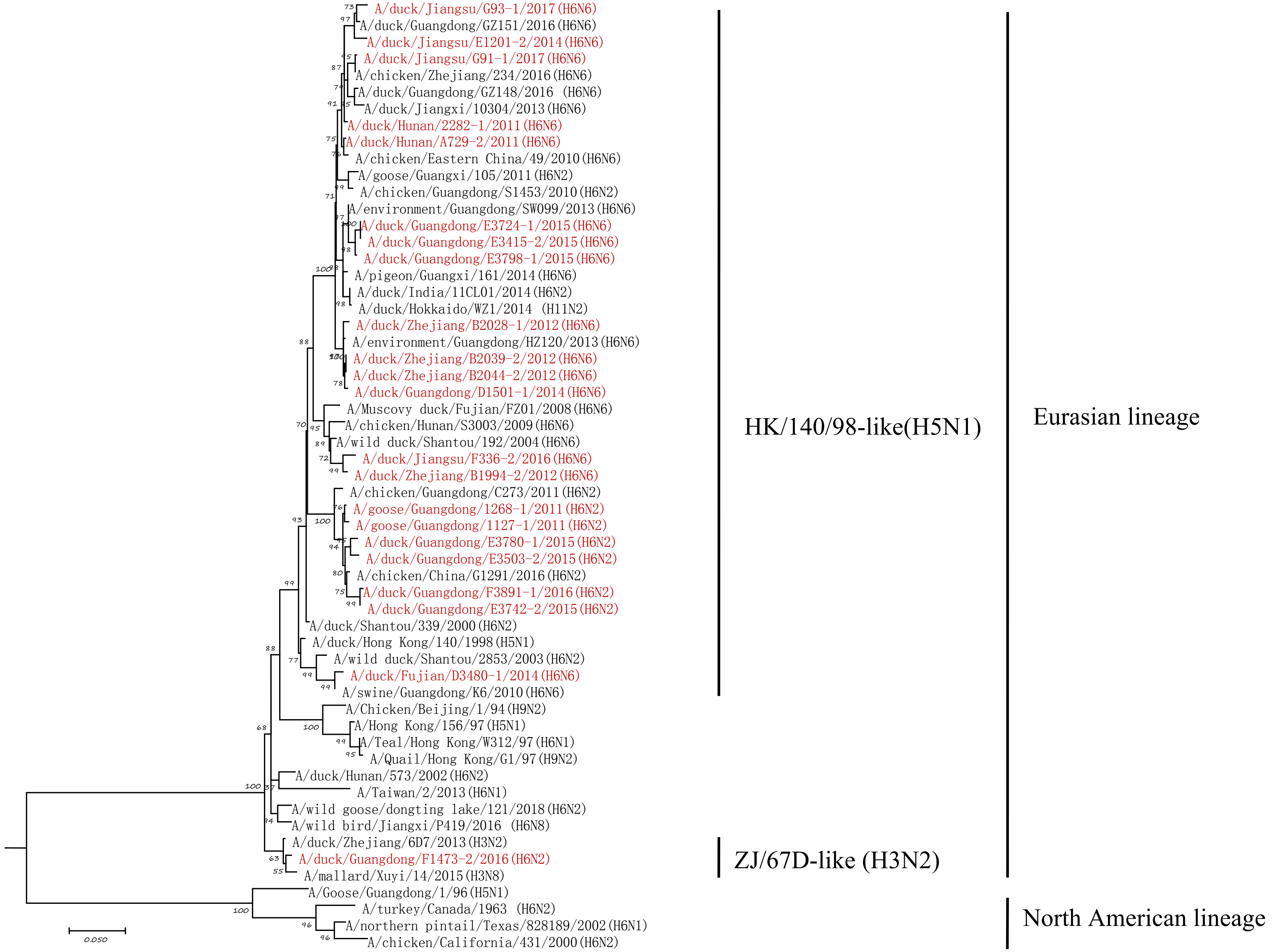

Fig. S6 Phylogenetic trees of NS of H6 AIVs. The phylogenetic tree was generated by the neighbor-joining method using MEGA 6.0. The viruses listed in black were downloaded from available databases; the viruses listed in red were evaluated in this study. The scale bar represents the distance between sequence pairs, and horizontal distances are proportional to genetic distance.
